# Assessment of Risk Factors and Prognostic Predictors for Endo-Perio Lesions in Indian Cohorts: An Observational Study

**DOI:** 10.7759/cureus.69598

**Published:** 2024-09-17

**Authors:** Lalitha B Shiggaon, Amar Kingaonkar, Tarundeep Kour, Saurabh Bhavsar, Malik Ayaz, Ankush Chaudhary, Seema Gupta

**Affiliations:** 1 Department of Periodontology, Jawahar Medical Foundations Annasaheb Chudaman Patil Memorial Dental College, Dhule, IND; 2 Department of Oral Pathology, Chhatrapati Shahu Maharaj Shikshan Sanstha Dental College and Hospital, Chhatrapati Sambhajinagar, IND; 3 Department of Dentistry, Sri Maharaja Gulab Singh Hospital, Jammu, IND; 4 Department of Conservative Dentistry and Endodontics, Kothiwal Dental College and Research Centre, Moradabad, IND; 5 Department of Dentistry, Arora Dental Care, Kapurthala, IND; 6 Department of Orthodontics, Kothiwal Dental College and Research Centre, Moradabad, IND

**Keywords:** endodontic, lesions, periodontal, predictors, risk factors

## Abstract

Introduction: Endodontic and periodontal tissues exhibit a significant interrelationship, whereby pathologies affecting one tissue can precipitate the involvement of the other. The primary objective of this study was to assess the risk factors associated with endo-perio lesions (EPLs). The secondary objective was to assess the prognostic predictors for such lesions and the prevalence rate of EPLs involving both pulpal and periodontal tissues in Indian cohorts.

Materials and methods: A prospective observational study was conducted on 2170 teeth with EPLs in Indian adults aged > 18 years. A detailed history of smoking, tobacco chewing, and the development of EPLs was obtained from all patients. Detailed periodontal and endodontic examinations were conducted by two calibrated examiners. Intraoral periapical radiographs (IOPAs) were obtained to diagnose EPLs. The chi-square test of association was used to analyze the association between independent and dependent variables, and the strength of association was presented by Pearson contingency coefficient (r). Logistic regression analysis was performed to identify reliable factors for diagnosing EPLs. A statistical significance threshold of 0.05 was established.

Results: The prevalence of EPLs involving pulpal and periodontal tissues was 29.49%. Type 1 lesions (EPLs involving only pulpal tissue) were most commonly seen at 41-45 years of age, type 2 lesions (EPLs involving primary pulpal and secondary periodontal tissue), and type 3 lesions (EPLs involving only periodontal tissue) at 26-30 years of age, and type 4 lesions (EPLs involving primary periodontal and secondary pulpal tissue) at 31-35 years of age. The pulp was found to be non-vital in all cases of type 3 lesions, and faulty restoration or improper root canal treatment (RCT) was the main risk factor for such lesions. Occupational trauma was found to be the most common risk factor for type 3 and type 4 lesions.

Conclusion: Smoking, age, tooth type, occlusal trauma, faulty restorations and RCT, presence of caries, periapical pathology, periodontitis, bone loss, and pulp vitality were significantly associated with EPLs. Pulp vitality and bone loss were two prognostic predictors of EPLs.

## Introduction

The pulpal tissue and periodontium exhibit a profound interrelationship, whereby pathologies affecting one tissue may precipitate complications in the other. Differentiating between endodontic and periodontal pathologies can occasionally pose challenges; however, it is imperative to achieve an accurate diagnosis to ensure the provision of suitable therapeutic interventions. Endo-perio lesions (EPLs) present significant obstacles for clinicians regarding the diagnostic accuracy and prognostic evaluation of affected dental structures [1]. The transfer of bacterial populations from one anatomical site to another is also possible, and this phenomenon can transpire in both directions (e.g., from the root canal to the periodontium, or conversely) via various communication pathways, including the apical foramen, lateral canals, and accessory canals, as well as dentinal tubules, developmental anomalies, and other pathological or iatrogenic alterations of the tooth root (such as carious lesions or fractures) [2].

According to the 2017 World Workshop on the Classification of Periodontal and Peri-implant Diseases, the prevalence of EPL lesions was 4.9% [3]. It was reported to be 14.89% of Indian populations by Altaf et al. [4]. The interaction between pulpal and periodontal tissues is very complex, and because of the involvement of various factors, correct recognition of the etiological factor is of utmost importance for the successful management of the case. The pathophysiology of true-combined lesions resembles that of primary endodontic and periodontal lesions. It is constituted by the confluence of two distinct lesions: the periapical lesion arising from pulpal necrosis, and the periodontal lesion that advances apically. This complicates the diagnostic process as an individual lesion may exhibit characteristics indicative of both endodontic and periodontal pathologies.

A novel classification of the interrelationship between endodontic and periodontal diseases, predicated on the primary condition and its subsequent ramifications, was proposed by Al-Fouzan, which was as follows: retrograde periodontal disease subdivided as primary endodontic lesion exhibiting drainage via the periodontal ligament, and primary endodontic lesion accompanied by secondary periodontal involvement; primary periodontal lesion; primary periodontal lesion associated with secondary endodontic involvement; concomitant endodontic-periodontal lesion; and lastly iatrogenic periodontal lesions [5]. Due to the lack of sufficient studies on the risk factors and predictors of EPLs, this study was conducted to assess the risk factors of EPLs in Indian cohorts as the primary objective, and the secondary objective was to assess the predictors of EPLs using logistic regression analysis.

## Materials and methods

Study design and setting

This prospective, observational, cross-sectional study was conducted in the Department of Periodontology, Jawahar Medical Foundation’s Annasaheb Chudaman Patil Memorial Dental College, from January 2023 to March 2024 after obtaining ethical approval from the institutional ethical committee (EC/NEW/INST/2021/2959/2023/163). The study followed the Strengthening the Reporting of Observational Studies in Epidemiology (STROBE) guidelines and principles of the Declaration of Helsinki. Written informed consent was obtained from all patients.

Sample size calculation and patients’ eligibility

The sample size was calculated using G power version 3.6, based on a reference prevalence rate of 17% for EPLs, as reported in previous studies [6]. To ensure sufficient statistical power, a 95% confidence interval and a margin of error of 5% were used for sample size determination. Given these parameters, the required sample size was calculated as approximately 2170 teeth. Patients older than 18 years and those with both periodontal and pulpal involvement were included in the study. Patients with systemic disease, those taking corticosteroids or long-term antibiotics, pregnant and lactating females, and patients with known allergic conditions were excluded from the study. Only patients with a complete history of EPLs were included in the study.

Methodology

The demographic details of the patients were noted, such as age, sex, history of smoking or chewing tobacco, and details on pulpal and periodontal problems. If the detailed history of the patient revealed primary pain in the tooth due to caries followed by tooth mobility, then it was indicative of a primary pulpal lesion with secondary periodontal involvement, whereas if the patient had a history of primary tooth mobility, pain on biting, followed by pulpal involvement, it was indicative of a primary periodontal lesion with secondary pulpal involvement. Intraoral periapical radiographs (IOPA) of the patients were obtained using the parallax technique to assess the involvement of the pulpal and periodontal tissues. In the case of primary periodontal involvement, the IOPA would show vertical or horizontal bone loss, interdental or interradicular radiolucency with the presence of periodontal pocket, and mobility on clinical examination, whereas in the case of primary pulpal involvement, IOPA would show a well-defined periapical radiolucency with or without the presence of a periapical abscess. Pulp vitality tests (cold and electric pulp testing) were performed to check vitality in the case of deep carious lesions. The cold testing was performed by applying a cold stimulus to the tooth using substances such as Endo Ice (1,1,1,2-tetrafluoroethane, Norflurane, Gurugram, India). Electric pulp testing was done with an electric pulp tester (Kerr Endodontics, Envista Holdings Corporation, Brea, California). If the tooth responded to the pulp vitality test, it indicated that the lesion was primary periodontal tissue. A non-vital tooth with significant periapical radiolucency may indicate a primary pulpal lesion. The periodontal condition was assessed using the modified periodontal index by Russel [7]. Simon’s classification was used to identify EPLs as primary endodontic lesions (type 1), primary endodontics with secondary periodontal involvement (type 2), primary periodontal lesion (type 3), primary periodontal with secondary endodontic involvement (type 4), and true-combined lesion (type 5) [8].

The endodontic examination was performed by one calibrated examiner, and similarly, the periodontal examination was performed by one calibrated examiner, who was calibrated on ten patients who were not involved in the study. The kappa test showed an excellent reliability of 90.3% for categorical data, and the intraclass correlation coefficient (ICC) test showed an excellent reliability of 92% for continuous data.

Statistical analysis

All data were analyzed for normal distribution using the Shapiro-Wilk test. Comprehensive descriptive data analysis was conducted to ascertain the basic characteristics of the sample. Means and standard deviations were computed for continuous variables, while frequency distributions were generated for categorical variables. The chi-square test of association was used to analyze the association between independent and dependent variables, and the strength of association was presented by Pearson contingency coefficient (r). Variables with frequency values less than five in two or more cells were analyzed using Fisher’s exact test. Logistic regression analysis was performed to identify reliable factors for the diagnosis of EPLs. A statistical significance threshold of 0.05 was established, and the data were analyzed using SPSS software (IBM Corp. Released 2013. IBM SPSS Statistics for Windows, Version 22.0. Armonk, NY: IBM Corp.).

## Results

Analysis of the study sample revealed that the prevalence of EPLs involving both pulpal and periodontal tissues (types 2 and 4) was 29.49%. Type 1 lesions were the most common in 880 (40.55 %) teeth, followed by type 3 in 650 (29.95%) teeth, type 2 in 370 (17.05%) teeth, and the least common was type 4 in 270 (12.44%) teeth. Type 5 was not found in our study sample; therefore, it was not included in our analysis. Type 1 lesions were most commonly seen at 41-45 years of age, type 2 and type 3 at 26-30 years of age, and type 4 at 31-35 years of age. This showed that involvement of both pulpal and periodontal tissues was noticed at 25-35 years of age. There was a statistically significant weak association between age and the presence of EPLs (P =0.011). However, gender did not show a significant association with EPLs, although EPLs were more commonly seen in males than in females.

There was a statistically significant weak association between smoking and the presence of EPLs (p=0.002), though 1420 teeth (65%) were present in non-smokers. Type 1 and 2 lesions were present predominantly in patients who had a habit of tobacco chewing, whereas type 3 and 4 lesions were predominantly in non-tobacco chewers, and there was no significant association between tobacco chewing and EPLs. The mandible is more commonly involved than the maxillary jaw. The most commonly involved teeth were the molars, followed by the premolars, incisors, and canines. There was a statistically significant weak association between the number of involved teeth and the presence of EPLs (p=0.019).

Occlusal trauma due to high restorations, and plunger cusps, was most commonly seen at etiological factor in type 3 and 4 lesions, and there was a strong association between this factor and the presence of EPLs (p=0.001). Faulty poorly done restorations were most commonly noticed as etiological factors for type 1 and 2 lesions, and there was a moderate association between this factor and the presence of EPLs (p=0.001). The pulp was vital in 500 (23.04%) teeth with type 1 lesions, non-vital in 370 (17.05%) teeth with type 2 lesions, where all the involved teeth were non-vital, vital in 650 (29.95%) teeth with type 3 lesions, where all the involved teeth were vital, and 170 (7.83%) teeth had vital pulp with type 4 lesions. There was a strong positive association between tooth vitality and the EPLs (p=0.001).

Type 1 lesions showed enamel caries in 140 (6.45) teeth, dentinal caries in 360 (16.59%) teeth, and pulpal involvement in 380 (17.51%) teeth. Type 2 lesions included all teeth with pulpal involvement, whereas type 4 lesions showed no carious involvement. Type 3 lesions showed enamel caries in 170 (7.83%) teeth. There was a strong statistically significant association between the presence of caries, periapical lesions, bone loss, and EPLs (p=0.001). Type 1 lesions showed no periapical infection in 500 (23.04%) teeth, periapical abscess in 170 (7.83%) teeth, and periapical granuloma in 190 (8.76%) teeth. Type 2 lesions showed acute and chronic periodontitis, whereas type 3 and 4 lesions showed periodontal abscesses in 200 (9.22%) teeth and 110 (5.07%) teeth, respectively. Bone loss was not present in type 1 lesions, whereas type 3 lesions showed predominantly horizontal bone loss and type 4 lesions showed vertical bone loss (Table 1).

**Table 1:** Descriptive analysis and association of risk factors with endo-perio lesions (EPLs) using chi-square test. *Chi-square test **p<0.05: significant ^†^Fisher’s exact test Very weak correlation: 0.0<∣r∣<0.20; weak: 0.2≤∣r∣<0.4; moderate: 0.4≤∣r∣<0.6; strong: 0.6≤∣r∣<0.8; very strong: 0.8≤∣r∣≤1. Data presented in the form of n%

Variables	Category	Distribution of EPLs				P-Value	Pearson Contingency Coefficient (r)
		Type 1 n%	Type 2 n%	Type 3 n%	Type 4 n%		
		880 (40.55)	370 (17.05)	650 (29.95)	270 (12.44)		
Age group^†^ (years)	20-25	180 (8.29)	60 (2.76)	120 (5.53)	0 (0)	0.011**	0.38
	26-30	130 (5.99)	120 (5.53)	190 (8.76)	20 (0.92)		
	31-35	170 (7.83)	40 (1.84)	60 (2.76)	100 (4.61)		
	36-40	140 (6.45)	40 (1.84)	100 (4.61)	70 (3.23)		
	41-45	260 (11.98)	110 (5.07)	180 (8.29)	80 (3.69)		
Gender*	Male	460 (21.2)	190 (8.76)	350 (16.13)	130 (5.99)	0.986	0.05
	Female	420 (19.35)	180 (8.29)	300 (13.82)	140 (6.45)		
Smoking*	No	630 (29.03)	310 (14.29)	320 (14.75)	160 (7.37)	0.002**	0.36
	Yes	250 (11.52)	60 (2.76)	330 (15.21)	90 (4.15)		
Chewing tobacco*	No	500 (23.04)	200 (9.22)	300 (13.82)	130 (5.99)	0.587	0.13
	Yes	380 (17.51)	170 (7.83)	350 (16.13)	140 (6.45)		
Jaw*	Maxilla	380 (17.51)	120 (5.53)	240 (11.06)	70 (3.23)	0.366	0.17
	Mandible	500 (23.04)	250 (11.52)	410 (18.89)	200 (9.22)		
Tooth involved^†^	Incisor	200 (9.22)	40 (1.84)	150 (6.91)	60 (2.76)	0.019**	0.33
	Canine	60 (2.76)	0 (0)	0 (0)	00 (0)		
	Premolar	200 (9.22)	140 (6.45)	130 (5.99)	20 (0.92)		
	Molar	420 (19.35)	190 (8.76)	370 (17.05)	190 (8.76)		
Occlusal trauma*	Yes	40 (1.84)	40 (1.84)	280 (12.9)	180 (8.29)	0.001**	0.72
	No	840 (38.71)	330 (15.21)	370 (17.05)	90 (4.15)		
Faulty restoration and root canal treatment*	Yes	160 (7.37)	220 (10.14)	120 (5.53)	20 (0.92)	0.001**	0.56
	No	680 (31.34)	150 (6.91)	530 (24.42)	250 (11.52)		
Pulp vitality^†^	No	380 (17.51)	370 (17.05)	0 (0)	170 (7.83)	0.001**	0.81
	Yes	500 (23.04)	0(0)	650 (29.95)	100 (4.61)		
Caries^†^	No caries	00 (0)	0(0)	480 (22.12)	270 (12.44)	0.001**	0.85
	Enamel caries	140 (6.45)	0(0)	170 (7.83)	0 (0)		
	Dentinal caries	360 (16.59)	0(0)	0 (0)	0 (0)		
	Pulp involvement	380 (17.51)	370 (17.05)	0 (0)	0 (0)		
Periapical lesion^†^	No lesion	500 (23.04)	0(0)	0 (0)	0 (0)	0.001**	0.82
	Acute periodontitis	20 (0.92)	140 (6.45)	130 (5.99)	20 (0.92)		
	Chronic periodontitis	0 (0)	160 (7.37)	320 (14.75)	140 (6.45)		
	Periapical abscess	170 (7.83)	20 (0.92)	0 (0)	0 (0)		
	Periapical granuloma	190 (8.76)	0(0)	0 (0)	0 (0)		
	Periodontal abscess	0(0)	50 (2.3)	200 (9.22)	110 (5.07)		
Bone loss^†^	No bone loss	880 (40.55)	130 (5.99)	0 (0)	0 (0)	0.001**	0.87
	Vertical bone loss	0 (0)	220 (10.14)	270 (12.44)	200 (9.22)		
	Horizontal bone loss	0 (0)	20 (0.92)	380 (17.51)	70 (3.23)		

**Figure 1:**
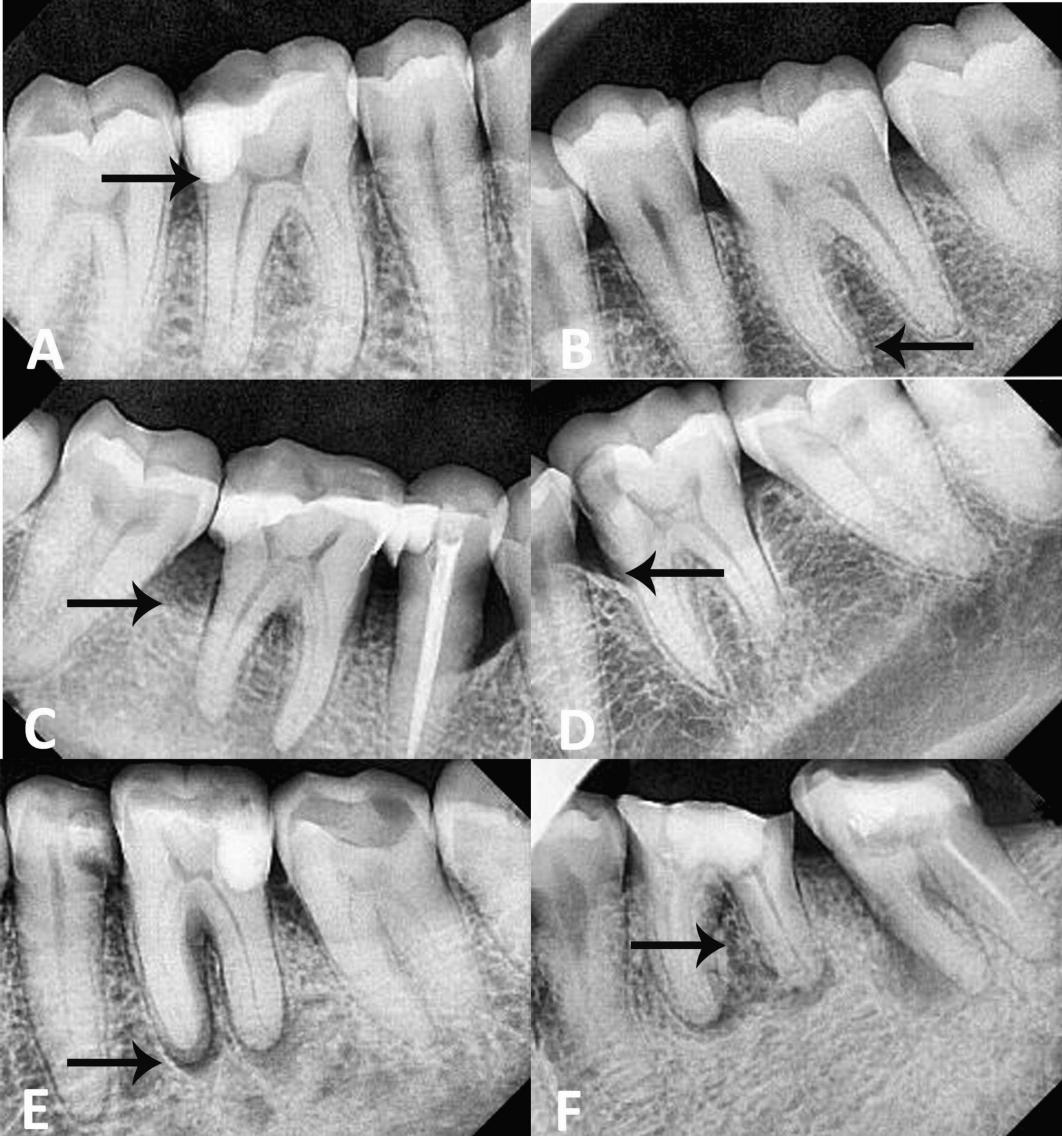
Types of endo-perio lesions (A) type 1 endo lesion, (B) type 3 perio lesion, (C) type 2 (primary endo and secondary perio lesion) with horizontal bone loss, (D) type 4 (primary perio and secondary endo lesion) with vertical bone loss, (E) type 2 lesion with periapical pathology, and (F) type 4 lesion with periodontal abscess.

The types of EPLs are shown in Figure 1.

Pulp vitality was the variable that was most likely associated with EPLs and showed a very high odds ratio of 260.15, which was highly significant (p=0.001). Bone loss also showed a strong association; however, the odds ratio was lower than that for pulp vitality. Therefore, vital pulp and bone loss were predictors of EPLs (Table 2).

**Table 2:** Logistic regression analysis for predictors of endo-perio lesions. *p<0.05: significant

Variables	Coefficient B	P-Value	Odds Ratio	95% Confidence Interval (Lower Limit-Upper Limit)
Age	0.16	0.129	2.23	1.78-4.67
Smoking	0.23	0.632	1.26	0.49-3.23
Chewing tobacco	-0.11	0.801	0.89	0.37-2.17
Periodontal index	-0.28	0.282	0.76	0.46-1.26
Pulp vitality	5.56	0.001*	260.15	26.31-2571.93
Caries	-2.04	0.079	0.13	0.01-1.27
Periapical pathology	-0.60	0.662	0.55	0.04-8.03
Bone loss	3.79	0.001*	44.34	8.19-240.14

## Discussion

The results of this study indicated that the most common reason for seeking dental treatment was dental pain due to caries; therefore, the most common EPL in our study sample was primary pulpal involvement. However, most of them sought dental care very late. This finding is in agreement with the study by Sachedina et al. [9]. The prevalence rate of dual involvement of the pulpal and periodontal tissues was 29.94% in our sample.

It has been noticed in our study that primary periodontal lesions (type 3) were mainly present at 26-30 years of age and 41-45 years of age. The presence of increased periodontal problems in the younger age group could have been due to smoking, which is significantly associated with EPLs. Cigarette consumption influences the metabolic processes of the alveolar bone and, consequently, may interact synergistically with alveolar bone resorption. Nicotine has the potential to induce alveolar bone resorption, vascular dilation, hemorrhagic events, degeneration of periodontal structures, and necrotic changes [10].

Type 3 and 4 lesions did not show involvement of pulpal tissues due to carious involvement and no periapical pathology. The pulp was vital in all type 3 lesions and in 63% of the teeth with type 4 involvement. This showed that deep cavitation is not present in peri-endo lesions (type 4) and therefore requires primary periodontal management followed by endodontic treatment [11]. Perio-endo lesions may occur as a result of anatomical irregularities, including palatogingival grooves, which promote bacterial infiltration without causing profound cavitation. Such grooves can penetrate to different depths, potentially establishing communication with the pulp chamber; however, they do not consistently lead to considerable cavitation.

Occupational trauma was found to be the most common risk factor for type 3 and type 4 lesions, highlighting the need for occlusal equilibration in such cases. The molars were the most commonly affected teeth, particularly in males. A similar finding was reported in a case report by Dwiyanti [12], in which a 63-year-old patient reported dull pain and periodontal abscess in the upper left molar due to excessive forces by an ill-fitting denture and trauma from occlusion leading to perio-endo lesions.

The pulp was non-vital in all cases of type 3 lesions, necessitating primary endodontic treatment followed by periodontal management. Faulty restoration or improper root canal treatment (RCT) is the main risk factor for such lesions. Faulty restorations may permit the infiltration of bacteria and toxins into the interstice between the restoration and the tooth, resulting in caries and infection. This phenomenon can adversely affect the integrity of tooth structure and adjacent periodontal tissues. In instances where the root canal system is inadequately cleaned and shaped, or if there is inadvertent perforation of the root during the procedure, it may facilitate the dissemination of residual bacteria or necrotic tissue to neighboring periodontal tissues [13].

The results of this study indicate that pulp vitality and bone loss are two predictors of an EPL diagnosis. In type 3 lesions, the pulp vitality test was negative in all cases, showing a non-vital pulp with the presence of vertical bone loss, whereas in type 4 lesions, the pulp may or may not be vital, with no carious involvement of teeth. Pulpal involvement is due to the seepage of bacteria from the lateral or accessory canals into the pulpal chamber. The loss of cementum due to previous root planning leads to the exposure of the dentinal tubules. The accumulation of bacterial biofilm on exposed root surfaces, subsequent to periodontal disease, can elicit pathological alterations within the pulp via lateral or accessory canals [14]. Moreover, there was an absence of any periapical pathology in type 4 lesions, where periodontitis with vertical bone loss was observed in most cases. Similar results have been reported by Guo et al. [15].

Limitations

This study was conducted in specific regions of India, and the findings might not be generalizable to the entire Indian population or to other ethnic groups. Variability in the diagnostic criteria for EPLs could have led to misclassification, affecting the study outcomes. EPLs have multifactorial causes, and controlling for all potential confounders such as oral hygiene maintenance and dietary habits can be challenging. Residual confounding could have affected the study’s conclusions. The study relied on self-reported data for factors such as smoking, tobacco chewing, and complete history of EPL development, which might lead to inaccuracies due to recall bias or social desirability bias.

## Conclusions

The results of this study indicated that EPLs involving only pulpal tissues were the most common lesions observed in the study sample. Smoking, age, tooth type, occlusal trauma, faulty restorations and RCT, presence of caries, periapical pathology, periodontitis, bone loss, and pulp vitality were found to be significantly associated with EPLs. Pulp vitality and bone loss were two prognostic predictors of EPLs. Primary endodontic and secondary periodontal lesions were associated with non-vital pulp and periapical pathologies, whereas primary periodontal and secondary endodontic lesions were associated with non-carious teeth and no periapical pathology.
